# Genomic insights into the overlap between psychiatric disorders: implications for research and clinical practice

**DOI:** 10.1186/gm546

**Published:** 2014-04-28

**Authors:** Joanne L Doherty, Michael J Owen

**Affiliations:** 1 The MRC Centre for Neuropsychiatric Genetics and Genomics and The Neuroscience and Mental Health Research Institute, Cardiff University, Hadyn Ellis Buildin, Maindy Road, Cardiff CF24 4HQ, UK

## Abstract

Psychiatric disorders such as schizophrenia, bipolar disorder, major depressive disorder, attention-deficit/hyperactivity disorder and autism spectrum disorder are common and result in significant morbidity and mortality. Although currently classified into distinct disorder categories, they show clinical overlap and familial co-aggregation, and share genetic risk factors. Recent advances in psychiatric genomics have provided insight into the potential mechanisms underlying the overlap between these disorders, implicating genes involved in neurodevelopment, synaptic plasticity, learning and memory. Furthermore, evidence from copy number variant, exome sequencing and genome-wide association studies supports a gradient of neurodevelopmental psychopathology indexed by mutational load or mutational severity, and cognitive impairment. These findings have important implications for psychiatric research, highlighting the need for new approaches to stratifying patients for research. They also point the way for work aiming to advance our understanding of the pathways from genotype to clinical phenotype, which will be required in order to inform new classification systems and to develop novel therapeutic strategies.

## The overlap between psychiatric disorders: challenges to current nosology

Psychiatric disorders are common in the population [1] and result in considerable morbidity and mortality [2]. Over the decades, psychiatric classification systems such as the Diagnostic and Statistical Manual of Mental Disorders (DSM) [3] and the International Classification of Diseases (ICD) [4] have been developed and revised in order to improve the reliability of clinical diagnosis, inform treatment strategies and guide research. However, in the absence of objective diagnostic tests for psychiatric disorders, such classifications are largely descriptive and syndromic, describing constellations of symptoms and signs that tend to occur together. These classification systems thus define psychiatric disorders such as schizophrenia, bipolar disorder, major depressive disorder, attention-deficit/hyperactivity disorder (ADHD) and autism spectrum disorder (ASD) categorically, according to the quality and quantity of symptoms and signs present. Treatment guidelines have been developed on the basis of these diagnostic categories. For example, schizophrenia is treated with antipsychotics, bipolar disorder with mood stabilizers and antipsychotics, major depression with antidepressants and ADHD with psychostimulants (Table 1).

**Table 1:** Clinical features, age of onset, lifetime prevalence and pharmacological management of major mental disorders

**Disorder**	**Core features**	**Associated features**	**Typical age of onset (years)**	**Lifetime prevalence (%)**	**Pharmacological management**
ADHD	InattentionHyperactivityImpulsivity	Cognitive impairment	7 to 12	5 [5]	Psychostimulants (for example, methylphenidate)
ASD	Deficits in social communication and social interaction Restricted and repetitive behaviors	Cognitive impairment Hallucinations Delusions	<3	1 to 2 [6-8]	No recommended drug treatment Medication used to treat comorbidities if present
Schizophrenia	Hallucinations Delusions Disorganized speech or behavior Apathy Lack of emotional reactivity	Cognitive impairment Discrete episodes of elevated, irritable or agitated mood Episodes of low mood	16 to 30	0.7 [9]	Antipsychotics (for example, risperidone)
Bipolar disorder	Discrete episodes of elevated, irritable or agitated mood	Episodes of low mood Hallucinations Delusions	18 to 40	1 [10]	Mood stabilizers (for example, lithium)Antipsychotics (for example, olanzapine)
Major depressive disorder	Low mood Loss of interest or pleasure Lack of energy	Psychosis	20 to 45	12.5 [10]	Antidepressants (for example, citalopram)

*ADHD*, attention-deficit/hyperactivity disorder; *ASD*, autism spectrum disorder.

However, it is widely acknowledged that there is substantial heterogeneity within diagnostic categories and that the boundary between disorder and ‘normality’ is not always clear. Furthermore, many symptoms and signs overlap between disorder categories and patients often present with features of more than one disorder. In some cases, this overlap has been dealt with by recognizing diagnostic ‘interforms’ such as schizoaffective disorder [11], or by recognizing ‘comorbidity’, whereby patients are diagnosed with more than one disorder. Comorbidity is often obscured in research by the use of diagnostic hierarchies or exclusions. For example, until the publication of the most recent edition of the DSM (DSM-5), it was not possible to co-diagnose ASD and ADHD. However, it is estimated that 30 to 80% of children with ASD also have ADHD [12,13]. Given these diagnostic issues, it is perhaps unsurprising that neuroscientific advances have thus far failed to identify specific risk factors or biomarkers that map onto disorder categories on a one-to-one basis. Indeed, there is accumulating evidence supporting biological overlap between disorders, fuelling investigation into the underlying mechanisms.

Advances in genomic technology have been key to this paradigm shift in psychiatric research. These studies provide converging evidence across a number of different levels, supporting the hypothesis that genetic risk factors are shared between disorders and challenging the validity of the classification systems currently used in research and clinical practice. The evidence suggests that investigating pathways common across disorders may help us to understand the etiology of psychiatric illness. This could revolutionize our approach to the diagnosis and treatment of these complex disorders. Here, we review recent evidence from family and genomic studies, which support an overlapping and complex genetic architecture for psychiatric disorders and provide new avenues for further investigation of underlying mechanisms.

## Family studies

Twin, family and adoption studies have provided heritability estimates for the major psychiatric disorders, highlighting an important role for genetic factors in the etiology of mental disorders [11,14-18].

In the late 19th century, Emil Kraepelin, a prominent German psychiatrist, distinguished between ‘dementia praecox’ (later termed schizophrenia) and manic-depressive psychosis (later termed bipolar disorder) as the two most common functional psychoses. Many family studies seemed to support this so-called ‘Kraepelinian dichotomy’, finding no familial relationship between schizophrenia and bipolar disorder, suggesting that these disorders ‘breed true’ [19-22]. These studies typically used hierarchical main-lifetime diagnoses - the predominant diagnosis an individual is given - in which schizophrenia was ranked higher than bipolar disorder. Therefore, patients who had several manic or depressive episodes in the past but who mostly had symptoms of schizophrenia later in the course of their illness would be given a main-lifetime diagnosis of schizophrenia. However, when non-hierarchical approaches were used, familial co-aggregation between schizophrenia, bipolar disorder [23] and schizoaffective disorder [24] was observed. This overlap in familial risk was confirmed in a meta-analysis of family studies of schizophrenia and bipolar disorder [25]. Recent large-scale population studies in Swedish cohorts have expanded on this, showing that a family history of schizophrenia, mood disorders, ASD and ADHD is associated with cross-disorder risk [26,27]. The overlap between major depressive disorder and other mental disorders is less clear. A meta-analysis of family high-risk studies recently compared offspring of parents with schizophrenia, bipolar disorder and major depressive disorder [28]. The study showed that offspring of adults with schizophrenia, bipolar disorder or major depressive disorder had a 32% probability of developing one of these disorders themselves by adulthood and that risks to offspring were not limited to their parent’s index disorder. For example, offspring of patients with schizophrenia or bipolar disorder had an increased risk of schizophrenia, bipolar disorder and major depressive disorder. However, although the relative risk of schizophrenia and bipolar disorder was also elevated in offspring of parents with depression, this finding did not reach statistical significance.

Vandeleur and colleagues [29] recently studied families with mood disorders (schizoaffective disorder, bipolar disorder and major depressive disorder), taking both categorical (disorder-based) and dimensional (symptom-based) approaches. Interestingly, the authors found that psychotic, manic and depressive symptoms were transmitted independently, suggesting that studying familial transmission of symptom dimensions may help to dissect clinical phenotypes in order to better understand the differential effects of genetic and environmental risk factors.

It has long been known that schizophrenia is associated with cognitive impairment and that this often predates the onset of psychotic symptoms [30]. Family studies show that the offspring of parents with schizophrenia have worse cognitive function than the offspring of unaffected parents [31], and that cognitive impairment is associated with familial risk of schizophrenia [32]. Applying genetic modeling to a combined family and twin sample, Toulopoulou and colleagues [33] showed that a large proportion of the phenotypic correlations between schizophrenia and cognition are due to shared genetic effects.

Taken together, the evidence from family studies suggests that psychiatric disorders may be co-inherited but also suggests that independent genetic and environmental risk factors are likely to be important in determining the ultimate clinical phenotype.

## Genomic studies

The epidemiological studies discussed above used the genetic relationships between individuals to study the co-inheritance of psychiatric disorders. Advances in genomic technology, including array-based genome-wide association studies (GWAS), and exome-sequencing techniques, have allowed the potential genetic mechanisms underlying the overlap between psychiatric disorders to be investigated more directly. Like other complex diseases such as hypertension and diabetes, psychiatric disorders are not inherited in a Mendelian fashion but have a complex genetic architecture involving a spectrum of mutations from small DNA sequence variations to large chromosomal rearrangements. Not only do these mutations vary in their size, they also vary in their frequency and associated effect sizes. Below, we discuss recent evidence for overlap in genetic risk factors from GWAS of single nucleotide polymorphisms (SNPs), studies of copy number variants (CNVs) and exome-sequencing studies of single nucleotide variants (SNVs), insertions and deletions (indels).

### Single nucleotide polymorphisms

The introduction of genome-wide association platforms has enabled researchers to study large samples of patients and controls to identify common variants (minor allele frequency >0.01) with cross-disorder associations. The markers used in these studies are SNPs: points in the DNA sequence where the nucleotide base varies in the population. In a recent paper published by the Cross-Disorder Group of the Psychiatric Genomics Consortium (PGC), four SNPs were identified as being significantly associated with the five psychiatric disorders studied: schizophrenia, bipolar disorder, major depressive disorder, ASD and ADHD [34]. Two of the SNPs were located in genes encoding L-type voltage-gated calcium channel subunits (*CACNA1C* and *CACNB2*). *CACNA1C* has previously been reported as having genome-wide significant associations with schizophrenia, bipolar disorder and major depressive disorder [35-38], and therefore there is strong evidence implicating calcium channel signaling in the pathophysiology of mental disorders. Other loci with cross-disorder associations include *ANK3*[36,37], *ZNF804A*[39,40] and *NCAN*[41,42], all of which have been reported in GWAS of schizophrenia and bipolar disorder.

To date, GWAS of common SNPs have shown that individual SNPs have modest effect sizes (typical odds ratio <1.2). It is therefore hypothesized that many common SNPs act together to influence risk of psychiatric disorders. Schizophrenia GWAS data support a polygenic basis for the disorder and estimate that common SNPs explain approximately one-third of the total variation in liability to schizophrenia [43,44]. These data also show that genetic liability is substantially shared with bipolar disorder [43]. Polygenic score analysis of common variants has provided further evidence for shared genetic risk across disorders. This approach uses a set of the top risk-associated alleles from a case-control ‘discovery’ GWAS sample to generate an aggregate score that can be used to test for differences between cases and controls in an independent ‘target’ sample. Scores are assigned to each individual based on the number of risk alleles they carry, weighted by the effect size for each variant. These scores are weighted by the effect size for each variant. Using this approach, it has been shown that alleles that are overrepresented in schizophrenia cases are also overrepresented in cases of bipolar disorder [43] and ADHD [45]. However, schizophrenia alleles were not found to be overrepresented in an ASD cohort [46]. Aggregate polygenic risk scores in the PGC sample also showed cross-disorder effects, which were strongest for adult-onset disorders [34]. Furthermore, polygenic risk scores have been used to investigate the association between schizophrenia and cognitive impairment. The Cognitive Genomics Consortium reported that schizophrenia polygenic risk scores were correlated with cognitive ability: higher ‘schizophrenia load’ being associated with lower cognitive ability. The authors also calculated cognitive polygenic scores and found that patients with schizophrenia had more alleles associated with poor cognitive performance and fewer alleles associated with good cognitive performance than controls [47]. In another study, schizophrenia polygenic risk score was associated with lower cognitive ability at age 70 years and with greater cognitive decline between childhood and old age [48]. However, another recent study failed to find an association between polygenic risk for schizophrenia and IQ variation in either schizophrenia cases or controls [49].

An alternative method for investigating the SNP-based genetic overlap between disorders is to use a method known as genome complex trait analysis (GCTA) [50] to calculate SNP-based genetic correlations. This method estimates the variance explained by all SNPs for a complex trait rather than testing the association of any particular SNP to the trait. It assumes an additive model and therefore does not account for gene-gene interactions. Using this method, it has been estimated that common variants contribute between 17 and 29% to the variation in liability to mental disorders [51]. High correlation was found between schizophrenia and bipolar disorder; moderate correlation between major depressive disorder and schizophrenia, major depressive disorder and bipolar disorder, and major depressive disorder and ADHD; and a small but significant correlation between schizophrenia and ASD. Interestingly, no correlations were found between other pairs of disorders, including ASD and ADHD, bipolar disorder and ADHD, or schizophrenia and ADHD. This was unexpected given the considerable phenotypic overlap between these disorders and data from family, twin and linkage studies, which suggest shared genetic risk factors [27,52-55]. However, when compared with adult-onset disorders, ADHD and ASD cohort sizes were relatively small in this study and therefore it may have been underpowered to detect SNP-based correlations between these disorders. Furthermore, there is increasing evidence for the role of rare genetic variants such as CNVs in neurodevelopmental disorders and it is possible that these play a more significant role in the overlap of ADHD and ASD than common SNPs, although further studies of larger samples will be needed in order to adequately address this.

### Copy number variants

Despite the high heritability of mental disorders reported in family studies, GWAS findings to date suggest that common variants only explain a relatively small proportion (about a third) of the genetic variation in liability to psychiatric illness [56]. Furthermore, the lack of SNP-based genetic correlation between disorders such as ADHD and ASD suggests a role for other types of variant in the genetic architecture of mental disorders. There is increasing evidence that rare structural variants (those present in <1% of the population), such as CNVs, may also be important. Owing to their low frequency in the population, these variants are unlikely to be tagged by common SNPs and therefore bring independent information about the genetic etiology of psychiatric disorders.

Several studies have clearly demonstrated enrichment for rare CNVs in psychiatric and developmental disorders. For example, it has been shown that CNV burden is higher in patients with schizophrenia [57-59], ADHD [60,61], ASD [62], intellectual disability (ID) and developmental delay [63] than in healthy controls. Furthermore, CNV load and the ratio of *de novo* to inherited CNVs correlate with the severity of developmental disability [63].

The role of CNVs in mood disorders is less clear. Several studies have shown that patients with bipolar disorder have a low burden of rare CNVs [64-66]. However, singleton deletions and *de novo* CNVs have been found to occur at a higher rate in bipolar disorder cases than in controls, particularly in early onset cases [67,68]. The only genome-wide study of CNVs in major depressive disorder found that deletions >100 kilobases (kb) in size were significantly enriched in patients with recurrent depression but that such CNVs explained only 0.87% of the variance between cases and controls [69].

Microdeletions and duplications conferring risk of schizophrenia have been reported at a number of chromosomal locations (Table 2) affecting genes involved in numerous processes such as synaptic signaling, neuronal migration, neurotransmitter metabolism and myelination. These CNVs show incomplete penetrance and have pleiotropic effects, resulting in variable clinical phenotypes. ID, ASD, ADHD, and mood and anxiety disorders commonly occur, as do epilepsy, congenital malformations, facial dysmorphology and developmental delay. Furthermore, ‘control’ carriers of CNVs (that is, those without a diagnosis of a psychotic illness, ASD, ID or developmental delay) at a number of known schizophrenia risk loci, including chromosomes 15q11.2 and 16p11.2, have been shown to have cognitive impairment, the severity of which is intermediate between that observed in patients with schizophrenia and population controls [70].

**Table 2:** Copy number variants associated with schizophrenia, their penetrance for schizophrenia, and associations with other psychiatric disorders and intellectual disability

**Locus**	**Copy number change**	**Penetrance**	**Associations**	**References**
1q21.1	Deletion/duplication	5.2/2.9	ID, ASD, ADHD	[57,58,71-75]
2p16.3 (*NRXN1*)	Deletion	6.4	ID, ASD	[57,58,74,76-81]
3q29	Deletion	18.0	ID, ASD	[73-75,82]
7q11.2	Duplication	6.0	ID, ASD, ADHD, anxiety disorders	[73,74,83-86]
15q11.2	Deletion	2.0	ID, ASD, ADHD, OCD	[72-74,81,87-89]
15q11-13	Duplication	4.2	ID, ASD	[73,74,90-92]
15q13.3	Deletion	4.7	ID, ASD, ADHD	[57,72-75,93-96]
16p11.2	Deletion/duplication	2.6/8.0	ID, ASD, ADHD, mood disorders, anxiety disorders	[62,73,74,91,97-102]
16p13.11	Duplication	2.2	ID, ASD, ADHD	[61,73,74,81,103,104]
17q12	Deletion	4.0	ID, ASD	[73,74,105]
22q11.2	Deletion	12	ID, ASD, ADHD, mood disorders, anxiety disorders	[57,73,74,106-109]

*ASD*, autism spectrum disorder; ADHD, attention-deficit/hyperactivity disorder; *ID*, intellectual disability; *OCD*, obsessive-compulsive disorder.

Recent evidence suggests that CNVs may not only increase risk of psychiatric disorders, they may also exert protective effects. The 22q11.2 deletion is the strongest known genetic risk factor for schizophrenia. Recently, Rees and co-workers [110] found that the reciprocal duplication is less common in patients with schizophrenia than in controls. This implies that studying CNVs may also help us to understand mechanisms underlying resilience to mental disorders.

### Single nucleotide variants and indels

GWAS and array-based techniques have enabled the detection of small common (>1%) and large rare (<1%) mutations. The development of exome-sequencing platforms has enabled the identification of small rare coding mutations, which could not previously be detected using GWAS or array-based techniques. Using samples of trios (both parents and one child) and quads (both parents with two children), *de novo* gene-disrupting SNVs and indels have been found to occur at higher rates in probands with ASD [111-114] and ID [115,116], being present in up to 14% of ASD cases [113,114] and up to 55% of ID cases [115] in the sampled cohorts. Furthermore, mutation rates were found to correlate with increasing paternal [111-113] and maternal [111,112] age, which is in line with epidemiological evidence for the positive correlation between parental age and rates of disorders in offspring [117-120].

The first exome-sequencing studies in schizophrenia found higher than expected rates of non-synonymous *de novo* mutations in small samples of patients [121,122]. Two recent and much larger studies [123,124] did not observe an increased rate of either non-synonymous or loss of function *de novo* or inherited mutations in schizophrenia. However, enrichment of loss of function *de novo* mutations did occur in schizophrenia cases likely to have the greatest intellectual impairment (lowest scholastic attainment), although these did not include cases with diagnoses of severe intellectual impairment. Moreover, genes with *de novo* mutations in schizophrenia overlapped with those affected by *de novo* mutations in ASD and ID but not in controls, with loss of function mutations enriched even in the very small subset of genes (*N* = 7) with recurrent loss of function *de novo* mutations in ASD or ID [123]. These findings therefore demonstrate shared genetic overlap between schizophrenia, ASD and ID at the resolution not just of loci or even of individual genes, but also at the level of mutations with similar functional (loss of function) impacts. The authors also observed that the genes hit by *de novo* mutations and the mutation sites themselves showed the highest degree of evolutionary conservation (a proxy measure of functional importance) in ID, then in ASD, with least conservation seen in schizophrenia. These findings suggest that highly disruptive mutations play a relatively small role in schizophrenia, and also that the disorders differ by severity of functional impairment.

## Potential biological mechanisms for shared genetic risk

Recently, evidence for the convergence of genetic findings onto a coherent set of biological processes in psychiatric disorders has been accumulating. Results from GWAS [34,44], CNV [83,125] and sequencing studies [114,123,124] point to highly functionally related sets of post-synaptic proteins involved in neurodevelopmental processes, synaptic plasticity, learning and memory. These include L-type calcium channels, post-synaptic scaffolding proteins involved in NMDA (*N*-methyl-d-aspartate) signal transduction, proteins that interact with ARC (activity-regulated cytoskeleton-associated protein), referred to as the ARC complex [83], and brain-expressed genes that are repressed by fragile X mental retardation protein (FMRP) [123,126].

These findings are notable for their consistency across several studies using different designs, and for their convergence onto a set of biological processes involved in the regulation of synaptic plasticity, particularly at glutamatergic synapses. Although there appears to be a concentration on post-synaptic mechanisms, some implicated genes, including the L-type calcium channels and neurexin 1 (an associated CNV locus [127]) also exert effects on plasticity pre-synaptically. Furthermore, these synaptic genes have been implicated in cognition [128] as well as in a range of psychiatric disorders, including schizophrenia, bipolar disorder, ASD and ID. However, the degree to which the associated pathways cross current diagnostic boundaries remains to be fully established. It is highly unlikely that this will represent the only set of biological processes implicated in these disorders, but the identification of at least one system involved in risk for schizophrenia and related disorders paves the way for more detailed mechanistic studies and potentially for stratified and novel therapeutic approaches.

Convergent support for glutamatergic synaptic processes is encouraging, but as already mentioned, it is likely that other processes are involved. There is a highly convincing body of evidence implicating dopaminergic dysfunction in the genesis of psychotic symptoms, which occur commonly in schizophrenia and bipolar disorder but that also occur in other neurodevelopmental disorders [129]. Indeed, the mechanism of action of antipsychotic drugs is understood to largely depend upon the blockade of dopamine D2 receptors. Understanding the relationship between glutamatergic dysfunction, which is closely related to cognitive impairment, and dopamine abnormalities is likely to be a fruitful approach to understanding how psychosis arises in schizophrenia and related disorders.

It seems probable that genetic disruption of the synaptic processes implicated to date will have widespread impacts on brain function and impair information processing within local, regional and even whole-brain networks - for example, by affecting excitatory-inhibitory balance and/or synaptic plasticity. Therefore, it is not surprising that mutations in genes involved in these processes affect cognitive function irrespective of the presence of comorbid psychopathology [48,70].

**Figure 1:**
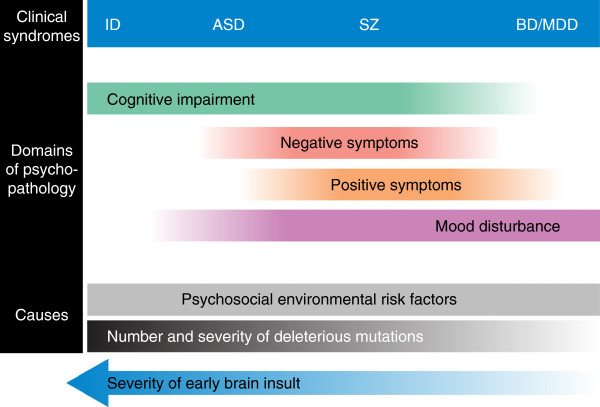
**Simplified representation of the hypothesized relationship between the number and severity of deleterious genetic mutations and clinical syndromes.** Psychiatric disorders as currently classified are shown as a neurodevelopmental continuum, with intellectual disability (ID) at one extreme and mood disorders at the other (see [130]). Domains of psychopathology overlap between the clinical syndromes, with the ultimate clinical phenotype being dependent on both genetic and environmental influences. Positive symptoms refer to abnormal thoughts, perceptions and behaviour, for example, hallucinations and delusions. Negative symptoms refer to disruption to normal emotions or behaviour, for example, apathy and lack of emotional reactivity. A gradient of mutational load and cognitive impairment is shown, with ID associated with the highest mutational load and most severe cognitive impairment, and mood disorders associated with the lowest mutational load/severity and least impaired cognitive function. The severity of individual syndromes is not represented. Owing to the lack of evidence from adequately powered genetic studies, attention-deficit/hyperactivity disorder (ADHD) has been omitted from the figure. ASD, autism spectrum disorder; BD, bipolar disorder; MDD, major depressive disorder; SZ, schizophrenia.

A spectrum of neurodevelopmental causality has previously been proposed to underlie the overlap between psychiatric disorders [130,131]. This proposed model suggests that psychiatric disorders and ID lie on a gradient of severity, with ID at one extreme and mood disorders at the other. Recent genomic evidence supports such a model and extends it to include a gradient of mutational severity or mutational load as well as cognitive impairment (Figure 1). Under such a model, genetic factors (such as the nature, quantity, size and location of deleterious genetic variants) and environmental factors leading to early brain insult act together to alter neurodevelopmental trajectories: the timing, severity, anatomical location and extent of the deviation from ‘normality’ determining the ultimate clinical phenotype. Further investigation, integrating genetic findings with cellular, animal, clinical and neuroimaging research, will shed more light on whether such a model is plausible.

## Implications for clinical diagnosis, management and research

Advances in our understanding of the genetic architecture of psychiatric disorders support the long-standing clinical observation of overlap in the symptoms and signs of these disorders, as well as the non-specificity of many environmental risk factors [131]. However, we are still some way off having sufficient new insights from genetics and neuroscience to replace current diagnostic approaches in the clinic. It is too early to say how advances in neuroscience and genetics will affect classification and diagnosis. Although current diagnostic categories are likely to remain clinically useful where they can best inform management and prognosis, these categories will require modification as further research indicates closer relationships of specific phenotypes and endophenotypes to mechanisms, and will probably need to include both dimensional (symptom-based) and categorical (syndrome-based) components.

What is clear, however, is that research into etiology and pathogenesis does require a radical overhaul in how we diagnose psychiatric illness, moving from categorical, hierarchical diagnoses based on consensus opinion towards a more dimensional approach informed by advances in our understanding of brain mechanisms. Indeed, such an effort is currently underway by the Research Domain Criteria (RDoC) project [132,133]. This project aims to provide the data required to build a new classification system based on underlying biology. While such a step is clearly needed, it will take a decade or more for enough evidence to be accumulated to develop a system that is suitable for clinical practice. However, an immediate implication of recent genetic findings and the RDoC project is the need to study patients who fall into the ‘gray area’ between disorder categories [134] or who have ‘subthreshold’ symptoms.

Pediatricians and clinical geneticists are increasingly using genetic testing for patients referred with congenital malformations, facial dysmorphology, developmental delay, ID and ASD [135,136]. So far, genetic testing has not been widely adopted in psychiatry (see [137] for an overview of some of the ethical issues involved in genetic testing in psychiatry). However, many CNVs associated with developmental abnormalities (for example, the 22q11.2 deletion) increase risk of mental health problems across the lifespan. A recent study by van den Bree and colleagues [138] found that the internet was the main source of parents’ information about psychiatric problems associated with 22q11.2 deletion syndrome, while another study reported that almost half of genetic counselors were uncomfortable discussing psychiatric disorders with affected families [139]. This suggests that there should be greater liaison between pediatricians, clinical geneticists and psychiatrists in order to appropriately counsel families about these risks and monitor symptoms.

There is now clear evidence that childhood-onset disorders often persist into adulthood [140,141] and that many adult-onset disorders have their roots in childhood [142,143]. Also, it is well established that cognitive impairment is a core feature across several disorder categories [144-147]. Psychiatric secondary care services are, for the most part, segregated into those that manage children and adolescents, adults of working age, older adults and patients with intellectual disability, respectively. Furthermore, neurodevelopmental disorders are often managed by other services, including clinical genetics, pediatrics and neurology. The evidence from recent genomic studies suggests that the service settings in which patients with neurodevelopmental disorders find themselves depend to a large extent on when, and by whom, they happen to be diagnosed, and that a lifetime neurodevelopmental perspective should be adopted in the diagnosis and management of psychiatric disorders and ID. Effective communication between service providers is key to providing high-quality care, particularly during transitions from one service to another.

One of the major barriers to improving the outcome of patients with severe mental illness is the lack of effective pharmacological treatments that are well tolerated by patients. As a result, psychiatric disorders are associated with high rates of treatment resistance and non-compliance due to poor drug efficacy and unacceptable side effects. Despite considerable advances in neuroscience, there have been few if any notable advances in pharmacotherapy, and as a consequence of this and the perceived challenges of the field, many pharmaceutical companies are discontinuing research in neuroscience and psychiatry [148]. Recent genetic advances have identified risk alleles, candidate genes and molecular pathways that could be exploited in the effort to identify novel drug targets and psychotherapeutic agents. However, such developments are going to need substantial investment in order to translate genetic findings from the laboratory into clinical practice.

In this article, we have emphasized the accumulating evidence from genetic studies suggesting shared susceptibility across traditional diagnostic categories in psychiatry. This implies that at least some of the underlying biology may not be specific, or at least not at the level of current diagnoses. However, it is important to recognize that relatively non-specific risk factors that apply to a wide variety of cases are easier to identify than those associated with more specific outcomes. There is evidence from both family and genomic studies for risk alleles with differential effects on schizophrenia and bipolar disorder [23,149] and also for alleles that have a degree of specificity to clinical phenotypes that do not necessarily relate well to traditional diagnostic categories [150]. Another example comes from the mounting evidence that large, rare CNVs that are more prevalent in schizophrenia are actually underrepresented in bipolar disorder [64,65]. Indeed, it seems highly probable that while psychiatric disorders, as currently defined, share risk genes and overlapping mechanisms, future research will identify risk alleles and mechanisms with more specific effects. The identification of risk alleles with greater phenotypic specificity will require researchers to tackle the problem of how to scale up detailed phenotyping to allow studies of the size required to detect genetic effects at robust levels of statistical significance. It also seems likely that we will have to move beyond cross-sectional descriptions of psychopathology and assemble data on course and outcome as well as endophenotypes linked to underlying neurobiological mechanisms. Recent history in psychiatric genetics tells us that these efforts will have to be large scale and collaborative.

## Conclusions and future directions

Clinical and epidemiological evidence for the overlap between psychiatric disorders has been available for some time. There is now increasing biological evidence for this overlap. Recent genetic findings suggest that psychiatric disorders have a complex genetic architecture and support the existence of a neurodevelopmental gradient of psychopathology and associated cognitive impairment. These findings highlight the need to improve current diagnostic systems and to develop better treatments targeted at underlying biology. Future research studying networks rather than individual genes and proteins, and relating network abnormalities to behavioral, neuroimaging and clinical phenotypes will be key to achieving these aims. However, the complexity of psychiatric phenotypes and the relative inaccessibility of the human brain pose considerable challenges to both basic and clinical neuroscientific research. It seems likely that there will need to be a focus on large-scale experiments, requiring interdisciplinary and international collaboration, if we are to make significant progress.

## Abbreviations

ADHD: Attention-deficit/hyperactivity disorder; ASD: Autism spectrum disorder; CNV: Copy number variant; DSM: Diagnostic and statistical manual of mental disorders; GWAS: Genome-wide association studies; ICD: International classification of diseases; ID: Intellectual disability; indel: insertion and deletion; kb: kilobases; PGC: Psychiatric genomics consortium; RDoC: Research domain criteria; SNP: Single nucleotide polymorphism; SNV: Single nucleotide variant.

## Competing interests

The authors declare that they have no competing interests.
